# Impact of Public Health and Social Measures on Cosmetic Treatments in the COVID‐19 Pandemic: A Retrospective Multi‐Center Study Combined With a Questionnaire‐Based Cross‐Sectional Study

**DOI:** 10.1111/jocd.16563

**Published:** 2024-09-24

**Authors:** Zichao Li, Tian Li, Leyang Zhang, Yifu Zhu, Zhou Yu, Baoqiang Song

**Affiliations:** ^1^ Department of Plastic Surgery, Xijing Hospital Fourth Military Medical University Xi'an China; ^2^ School of Basic Medicine Fourth Military Medical University Xi'an China

**Keywords:** anxiety states, cosmetic treatment, COVID‐19 pandemic, public health and social measures, questionnaire‐based study, time–series analysis

## Abstract

**Background:**

Public health and social measures (PHSMs) are considered the most effective approaches for controlling epidemic diseases. This study aimed to explore variations in the time‐dependent characteristics of and public preferences for cosmetic treatments during and after the implementation of PHSMs during the COVID‐19 pandemic.

**Method:**

Medical records from six medical institutions were extracted retrospectively. Time–series analyses were conducted to reveal the variations in characteristics in volume and proportion of cosmetic treatments according to PHSMs. A cross‐sectional study was conducted with online questionnaire designed for the general population during and after the implementation of PHSMs.

**Result:**

A total of 141 033 records were included in this retrospective study. The implementation of PHSMs led to extremely low treatment volumes; compared with the increases in private hospitals, the treatment volumes in public hospitals exhibited earlier and more significant increases, even higher than pre‐PHSM levels (*p* < 0.05), which mainly contributed to the increase in plastic surgery volumes during and after the implementation of PHSMs. The differences in the anxiety state, self‐perceived appearance, and cosmetic treatment intentions of the participants were illustrated during and after PHSMs. We further demonstrated the participants' decisions on cosmetic treatments after the implementation of PHSMs during the COVID‐19 pandemic.

**Conclusion:**

The immediate effects and aftereffects of PHSMs on cosmetic treatments were different in public and private hospitals. Furthermore, as PHSMs guided the adjustment of cosmetic treatments in the post‐COVID‐19 era, the intention to undergo cosmetic treatment during PHSMs was associated with the anxiety states and preferences of the population.

## 1 Introduction

The contemporary pursuit of self‐satisfaction and beauty typically increases the demand and desire for cosmetic treatments across the world [1]. Nevertheless, to slow the spread of the novel coronavirus following the outbreak of COVID‐19 in November 2019 [2], the government imposed necessary public health and social measures (PHSMs), which substantially influenced human behaviors, modes of transport, and patterns and plans of visit attendance of various medical appointments [3].

The COVID‐19 pandemic affected all aspects of medical practice, especially the delivery of elective cosmetic treatments [4]. According to the Federation of Surgical Specialty Associations in the UK [5], at the onset of the pandemic, since all cosmetic procedures fell into the lower levels of clinical priority (levels 1–4), a moratorium was placed on these elective treatments to prioritize emergency cases and urgent oncology treatment [6]. As COVID‐19 is characterized by high infectivity and aggregation, PHSMs were essential approaches to reduce the rate of coronavirus infection at the epidemic's peak [7]. Public and private hospitals took powerful action in terms of cosmetic treatment, as required by governments, such as forbidding the admission of patients without COVID‐19 infection and decreasing elective surgeries [8, 9].

Xi'an, a populous city in China, suffered a rapid spread of the imported Delta variant from December 24, 2021, to January 23, 2022, and most citizens practiced social distancing. The period of PHSM implementation not only greatly reduced the number of cosmetic procedures in hospitals but also affected the mental state and population's intentions of undergoing cosmetic treatments. However, no research was performed on evaluating the immediate effects and aftereffects of PHSM implementation during the COVID‐19 pandemic on the volume of cosmetic surgery procedures. Our retrospective multi‐center study and population‐based cross‐sectional study provided unique paradigms to investigate the time‐dependent variations associated with PHSMs in terms of cosmetic treatments in public and private hospitals and the population's demand for cosmetic treatments since the implementation of PHSMs; the findings will enhance our understanding of the development of cosmetic medicine during emergencies, such as disease pandemics and natural disasters.

## 2 Method

### 2.1 Data Sources and Classification of the Retrospective Study

To investigate the time–series characteristics of cosmetic treatments before, during, and after PHSMs during the COVID‐19 pandemic, we extracted 141 033 medical records from October 1, 2021, to May 30, 2022, from the high‐impact departments of plastic surgery and cosmetic dermatology in three public hospitals and three private hospitals of different sizes to ensure representativeness in the field of cosmetic medicine. Considering the time–series characteristics based on PHSMs, we divided the 8‐month timescale into the following five periods: October 1, 2021, to December 23, 2021 (before PHSMs); December 24, 2021, to January 27, 2022 (during PHSMs); January 28, 2022, to February 24, 2022 (1 month after PHSMs); February 25, 2022, to March 24, 2022 (2 months after PHSMs); March 25, 2022, to May 31, 2022 (normal pre‐COVID social behaviors stage). The recorded surgical and non‐surgical treatments approximately covered all cosmetic practices. Surgical treatments were defined as oculoplasty, otoplasty, rhinoplasty, mammaplasty, labiaplasty, maxillofacial surgery/facial osteoplasty, rhytidectomy, liposuction, facial lipofilling, abdominal wall reconstruction, hair transplantation, genital cosmetic surgery, and scar revision, while non‐surgical treatments were defined as laser and radiofrequency treatments, injections, and contour threading. Further exploration of the variations in the volumes and proportions of surgical and non‐surgical treatments in public and private hospitals was performed for the different periods. The acquisition and analysis of the data were approved by the ethics committee of Xijing Hospital of the Fourth Military Medical University (KY2022006‐D‐2).

### 2.2 Inclusion and Exclusion Criteria of the Participants

The cross‐sectional study was conducted to explore the public's desire to undergo cosmetic treatments of cosmetic treatments since PHSMs, which would improve the allocation of medical resources based on demand and supply in medical institutions. All participants in our questionnaire‐based study were residents of Xi'an, Shanxi Province, China. Informed consent regarding the purpose of the questionnaire was given by the participants. We borrowed a concept from the Condition, Context, and Population approach to establish our inclusion and exclusion criteria for participants during and after the implementation of PHSMs as a result of the COVID‐19 pandemic [10].

The participants in this study met the following four criteria: (1) Participants did not leave Xi'an from October 1, 2021 (initiation point) to May 31, 2022 (endpoint); (2) participants were at least 18 years of age; (3) participants understood the purpose of the study and voluntarily completed the self‐administered questionnaire; and (4) participants had a healthy mental state and a normal understanding of cosmetic treatments. Those with irrational demands or not suitable for cosmetic treatments distinguished by physicians clinically were excluded from our study.

### 2.3 Questionnaire Design and Delivery

Twenty‐five questions in five aspects were included in the questionnaire for the general population during and after the implementation of PHSMs due to the COVID‐19 pandemic (Table S1). First, items 1–5 collected the basic information of the participants. Then, items 6–11 systematically evaluated the anxiety state and self‐perception of appearance among the participants during and after PHSMs by referring to a self‐rating anxiety scale. Furthermore, for those participants planning to have cosmetic treatments, items 14 and 15 were designed to unveil the contribution of factors in choosing medical institutions and doctors during different periods. Finally, items 16–24 explored the public perceptions of expected recovery time, cost, and types of cosmetic treatments during the COVID‐19 pandemic. By pre‐testing the questionnaire, we determined the appropriate minimum time to complete all items to ensure the participants could read and fully understand the meaning of each question. The questionnaire was modified after three rounds and vetted by senior medical statisticians, plastic surgeons, and the ethics committee before delivery. The anonymous questionnaire was developed and delivered through the online platform https://www.wjx.cn/, and the personal identities of the responders were hidden to guarantee the privacy of all participants.

### 2.4 Reliability and Validity Assessments in the Questionnaire

Multiple measures were taken to ensure the reliability and validity of the questionnaire. The questionnaire's reliability was assessed by calculating the Cronbach's alpha value of items 6–9, which represented internal consistency. Coefficients with a value of ≥0.70 were considered highly reliable [11]. The construct validity of the questionnaire was established via an exploratory factor analysis based on principal component factoring [12]. Prerequisites for a further factor analysis were *p* < 0.05 in Bartlett's test of sphericity and a Kaiser–Meyer–Olkin (KMO) value of > 0.70 [13, 14], and factor loading values of ≥0.5 for the items were acceptable [15]. The submitted questionnaires were not regarded as valid unless they met the following three conditions: (1) The questionnaire was submitted between December 24, 2021, and January 23, 2022 (during PHSMs) or between January 24, 2022, and March 25, 2022 (after PHSMs); (2) all the questions should be completed in no less than 45 s; and (3) the participant completely understood all the questions.

### 2.5 Statistics and Data Visualization

Descriptive data were presented as frequencies and proportions. Chi‐squared tests were used to determine the differences in the proportions of the categorical variables, and Wilcoxon–Mann–Whitney tests and Kruskal–Wallis tests were conducted for differences in the ranked data. Additionally, *t* tests were used to compare the surgical volume between two different periods after testing the normality and homogeneity of variance of the data. A value of *p* < 0.05 was considered statistically significant. Radar plots and Nightingale rose diagrams were used to describe preferences in cosmetic treatments. The “ggradar” and “ggplot2” packages in R software (version 4.2.3) were used for data visualization. Resting statistical analyses were performed using Graph Pad Prism software (version 8.0).

### 2.6 Patient and Public Involvement

Patients or the public were not involved in the design, or conduct, or reporting, or dissemination plans of our research.

## 3 Results

### 3.1 Time–Series Curves of Cosmetic Treatments During the COVID‐19 Pandemic

**FIGURE 1:**
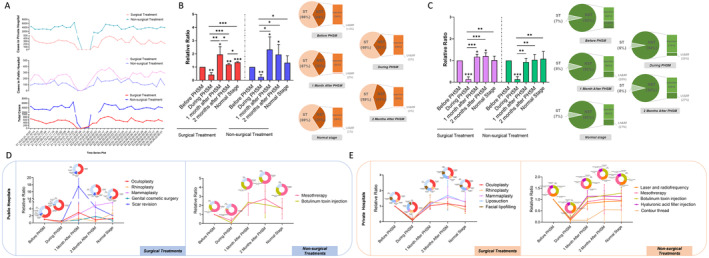
Time–series analysis of cosmetic treatments before, during, and after PHSMs. (A) Weekly volumes of surgical and non‐surgical treatments in public, private, and total hospitals are illustrated using line charts. (B) Relative ratios of average weekly volumes of surgical and non‐surgical treatments in public hospitals in different periods of PHSMs are presented in column charts, and proportions of surgical and non‐surgical treatments are illustrated in composite pie charts. (C) Relative ratios of average weekly volumes of surgical and non‐surgical treatments in private hospitals in different periods of PHSMs are presented in column charts, and proportions of surgical and non‐surgical treatments are illustrated in composite pie charts. (D) Relative ratios of time‐dependent variations in average weekly volumes and proportions of the top‐ranked treatments carried out jointly by three public hospitals represented in line and doughnut charts. (E) Relative ratios of time‐dependent variations in average weekly volumes and proportions of the top‐ranked treatments carried out jointly by three private hospitals represented in line charts and doughnut charts. NST, non‐surgical treatment; PHSMs, public health and social measures; ST, surgical treatment. *p < 0.05, **p < 0.01, ***p < 0.001.

The curves in Figure 1A systematically depict the time–series characteristics of cosmetic treatments in weekly units from October 1, 2021, to May 30, 2022. The involved surgical and non‐surgical treatments were classified in Table 1. The cosmetic treatments were delivered in both public and private hospitals due to the implementation of PHSMs on December 24, 2021; the volumes of cosmetic treatments exhibited a sharp decline and were sustained at extremely low levels until the fourth week of PHSMs (January 14–20, 2022). An obvious rise in treatment volumes up to a peak occurred in the third and fourth weeks after PHSMs (February 11–24, 2022), which were greater than those before the measures; this was followed by a gradual decline to a steady state. Due to differences in the composition of cosmetic treatments in public and private hospitals, the time–series curves of cosmetic treatments demonstrated different variation trends. In public hospitals, after the third week (January 7–13, 2022) of PHSMs, an ascending trend in treatment volumes occurred, especially for surgical treatments. However, cosmetic treatments in private hospitals were dominated by non‐surgical treatments, which gradually increased after the fifth week of PHSMs (January 21–27, 2022). In addition, it also took different times to reach the maximum volumes for surgical and non‐surgical treatments in public and private hospitals after the implementation of PHSMs (Figure 1A).

**TABLE 1:** Classification of cosmetic treatments.

	Public hospitals, *n* (%)	Private hospitals, *n* (%)	Total, *n* (%)
Surgical treatment			
Oculoplasty	4279 (53.40)	3240 (35.26)	7519 (43.71)
Otoplasty	220 (2.75)	0 (0.00)	220 (1.28)
Rhinoplasty	295 (3.68)	916 (9.97)	1211 (7.04)
Mammaplasty	163 (2.03)	339 (3.69)	502 (2.92)
Labiaplasty	94 (1.17)	32 (0.35)	126 (0.73)
Maxillofacial surgery facial osteoplasty	123 (1.54)	111 (1.21)	234 (1.36)
Rhytidectomy	17 (0.21)	4 (0.04)	21 (0.12)
Liposuction	56 (0.70)	1561 (16.99)	1617 (9.34)
Facial Lipofilling	135 (1.68)	914 (9.95)	1049 (6.10)
Abdominal wall reconstruction	15 (0.19)	0 (0.00)	15 (0.09)
Hair transplantation	57 (0.71)	132 (1.44)	189 (1.10)
Genital cosmetic surgery	148 (1.85)	57 (0.62)	205 (1.20)
Scar revision	303 (3.78)	743 (8.08)	1046 (6.08)
Other^a^	2108 (26.31)	1141 (12.42)	3249 (18.89)
Non‐surgical treatment			
Laser and radiofrequency	158 (3.83)	37 713 (31.5)	37 871 (30.58)
Mesotherapy	2380 (57.71)	29 096 (24.31)	31 476 (25.42)
Botulinum toxin injection	940 (22.79)	37 569 (31.38)	38 509 (31.10)
Hyaluronic acid filler injection	646 (15.66)	14 397 (12.03)	15 043 (12.15)
Collagen injection	0 (0.00)	609 (0.51)	609 (0.49)
Contour threading	0 (0.00)	322 (0.27)	322 (0.26)

^a^ Other: Other types of plastic surgery not included in the table.

### 3.2 Time–Series Analysis of Treatment Volumes and Proportions in Public Hospitals

In public hospitals, the average weekly volumes of surgical treatments 1 month after the implementation of PHSMs were higher than those in other groups, and the volumes after PHSMs were always higher than those before PHSMs (*p* < 0.05); however, for non‐surgical treatments, the average weekly volumes 1 and 2 months after PHSMs were significantly higher than those before and during the measures, and the volumes in the normal pre‐COVID social behaviors stage after PHSMs were equal to the volumes before PHSMs (Figure 1B). The proportions of surgical treatments and non‐surgical treatments usually maintained 66%–69% and 31%–34%, respectively, except for the 2 months after PHSMs, with 59% comprising surgical treatments and 41% comprising non‐surgical treatments; rises in the proportion of injection treatments mainly contributed to these differences. The differences in the average weekly treatment volume and proportion of the main treatments given in each public hospital in different periods are depicted in Figure 1D and Table S2.

### 3.3 Time–Series Analysis of Treatment Volumes and Proportions in Private Hospitals

Different characteristics in treatment volumes and proportions in distinct periods were found in private hospitals compared with those in public hospitals. The average weekly volumes of surgical treatments and non‐surgical treatments exhibited obvious rises after PHSMs, and the volumes of surgical treatments 1 and 2 months after PHSMs were statistically higher than those before PHSMs. No significant difference in the volume of non‐surgical treatments was revealed before and after PHSMs (Figure 1C). No significant fluctuation in the proportions of surgical and non‐surgical treatments was detected in different periods. However, comparing the proportions of injections and laser and radiofrequency in non‐surgical treatments at various stages, the proportion of injections increased from 60% before PHSMs to 79% during PHSMs, subsequently declining to 65%–66% after PHSMs; meanwhile, the proportion of laser and radiofrequency demonstrated a decline to varying degrees during and after PHSMs compared with the proportions before implementation (Figure 1C). The average weekly treatment volumes and proportions of some treatments with major proportions in private hospitals are presented in Figure 1E and Table S3.

### 3.4 Participant Characteristics in the Questionnaire‐Based Study

In addition to the retrospective multi‐center study, the cross‐sectional study was also necessary to understand public demands and preferences since the implementation of PHSMs (Table S1). We collected a total of 1040 questionnaires, of which 1018 (97.9%) responses were valid according to the inclusion and exclusion criteria, with 507 responses during PHSMs and 511 responses after PHSMs. Similar proportions of participants' baseline data (gender, age, marriage status, and salary) were recorded during and after PHSMs (*p* > 0.05), which implied that the datasets during and after the measures were comparable (Table 2). On this basis, higher average anxiety states were detected during PHSMs than after (603.48 vs. 416.26, *p* < 0.005). During PHSMs, participants had increased proportions of negative self‐perceived appearance and intentions to undergo cosmetic treatments, and higher proportions of participants increased their desire for undergoing cosmetic treatments (*p* < 0.05) (Table 2).

**TABLE 2:** Participants' characteristics in the questionnaire‐based study during and after PHSMs.

	PHSMs, *n* (%)	After PHSMs, *n* (%)	Total, *n* (%)	*p*‐value
Gender				0.17
Female	352 (69.43)	340 (66.54)	692 (67.98)	
Male	155 (30.57)	171 (33.46)	326 (32.02)	
Age (years)				0.64
18–30	342 (67.46)	340 (66.54)	682 (66.99)	
30+	165 (32.54)	171 (33.46)	336 (33.01)	
Marriage status				0.14
Married	188 (37.08)	206 (40.31)	394 (38.70)	
Unmarried	319 (62.92)	305 (59.69)	624 (61.30)	
Salary (CNY)				0.14
< 5000	251 (49.51)	237 (46.38)	488 (47.94)	
5000–10 000	161 (31.76)	160 (31.31)	321 (31.53)	
> 10 000	95 (18.74)	114 (22.31)	209 (20.53)	
Anxiety				< 0.001
No anxiety	168 (33.14)	353 (69.08)	521 (51.18)	
Mild anxiety	250 (49.31)	124 (24.27)	374 (36.74)	
Moderate anxiety	71 (14.00)	23 (4.50)	94 (9.23)	
Severe anxiety	18 (3.55)	11 (2.15)	29 (2.85)	
Self‐perceived appearance				< 0.001
Better	39 (7.69)	33 (6.46)	72 (7.07)	
Worse	161 (31.76)	135 (26.42)	296 (29.08)	
Unchanged	307 (60.55)	343 (67.12)	650 (63.85)	
CT intention				< 0.001
Yes	217 (42.80)	179 (35.03)	396 (38.90)	
No	290 (57.20)	332 (64.97)	622 (61.10)	
Change in CT intention				0.006
Increase	78 (15.38)	46 (9.00)	124 (12.18)	
Decrease	95 (18.74)	95 (18.59)	190 (18.66)	
No change	334 (65.88)	370 (72.41)	704 (69.16)	

Abbreviations: CNY, Chinese yuan; CT intention, intention to undergo cosmetic treatment.

### 3.5 The Reliability and Validity of the Questionnaire

Items 6 to 9 in the questionnaire demonstrated good internal consistency, with Cronbach's alpha = 0.92. For the exploratory factor analysis, both the KMO value (0.846) and Bartlett's test of sphericity (Chi‐squared statistic = 3045.495; *p* < 0.001) were statistically satisfied. One component was extracted based on an eigenvalue over 1, accounting for 80.73% of the total variance, which indicated that the questionnaire was a single‐dimension design. The factor loadings of items 6–9 were 0.878, 0.920, 0.918, and 0.878, respectively. Therefore, these results indicated the reliability and validity of our questionnaire.

### 3.6 The Impact of Anxiety States on the Intention to Undergo Cosmetic Treatment

Concerning the use of a self‐rating anxiety scale and the purpose of the study, items 6–9 schematically evaluated participants' anxiety states in terms of subjective feelings, somatic symptoms, and appearance anxiety during and after PHSMs (Table S8). On this basis, we found that participants' anxiety states significantly influenced their self‐perception of appearance (*p* < 0.05), and those participants who thought their appearance had worsened usually had more severe anxiety states (Table S8). Furthermore, a higher proportion of participants affected by PHSMs were more likely to consider that their appearance had deteriorated (*p* < 0.001), especially in terms of skin texture (*p* < 0.05), while no significant differences were manifested in the proportion of participants who considered their appearance had improved during and after PHSMs (Table S4). Moreover, different anxiety states were also closely related to differences in the intentions to undergo cosmetic treatments (*p* < 0.05). The average rank‐based scores indicated that participants with high intentions or increased intentions of undergoing cosmetic treatments might have had more severe anxiety states (Table S8).

### 3.7 Participants' Preferred Decisions About Cosmetic Treatments During the COVID‐19 Pandemic

**FIGURE 2:**
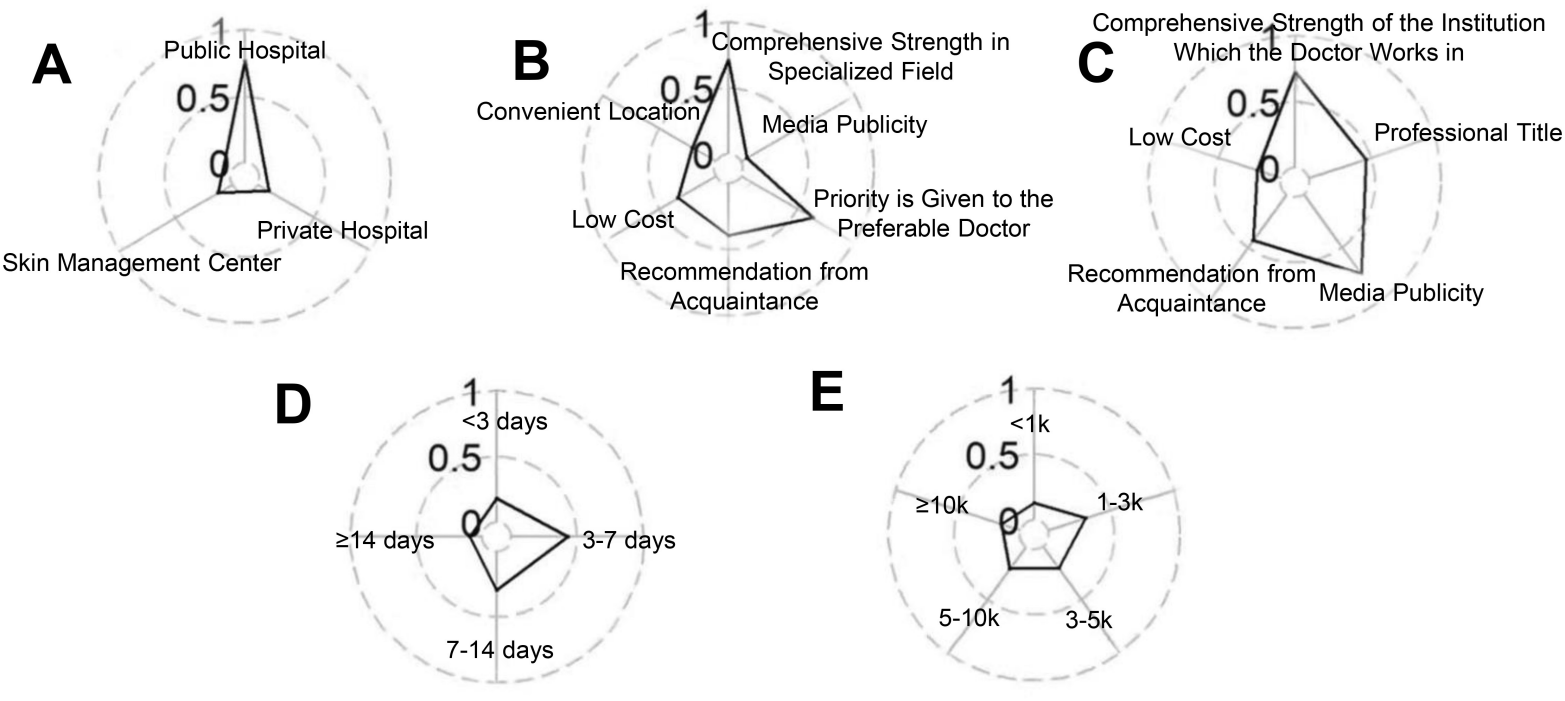
Profile of the proportion of participants' preferences for undergoing cosmetic treatments since the implementation of public health and social measures. (A) The preferred medical institutions of all participants with intentions of undergoing cosmetic treatments. (B) Factors influencing the choice of medical institutions for all participants with intentions of undergoing cosmetic treatment. (C) Factors influencing the choice of doctors for all participants with intentions of undergoing cosmetic treatment. (D) Expected recovery time of cosmetic treatments for all participants with intentions of undergoing cosmetic treatment. (E) Expected expenses of cosmetic treatment for all participants with intentions of undergoing cosmetic treatment.

We investigated the preferences and vital factors for receiving treatments in those who had intentions of undergoing cosmetic treatments after the implementation of PHSMs during the COVID‐19 pandemic. Most participants (77.80%) preferred to receive cosmetic treatment in public hospitals (Figure 2A). Over 60% of participants valued the hospitals that specialize in the field of cosmetic surgery and their preferred doctors in the hospital (Figure 2B). When choosing doctors, over 70% of participants valued the medical institution in which a doctor worked as well as doctors' professional titles (Figure 2C). Moreover, the expected recovery time of 3–7 days and the expected costs of 1000–3000 Chinese yuan (CNY) were acceptable to most people (Figure 2D,E).

**FIGURE 3:**
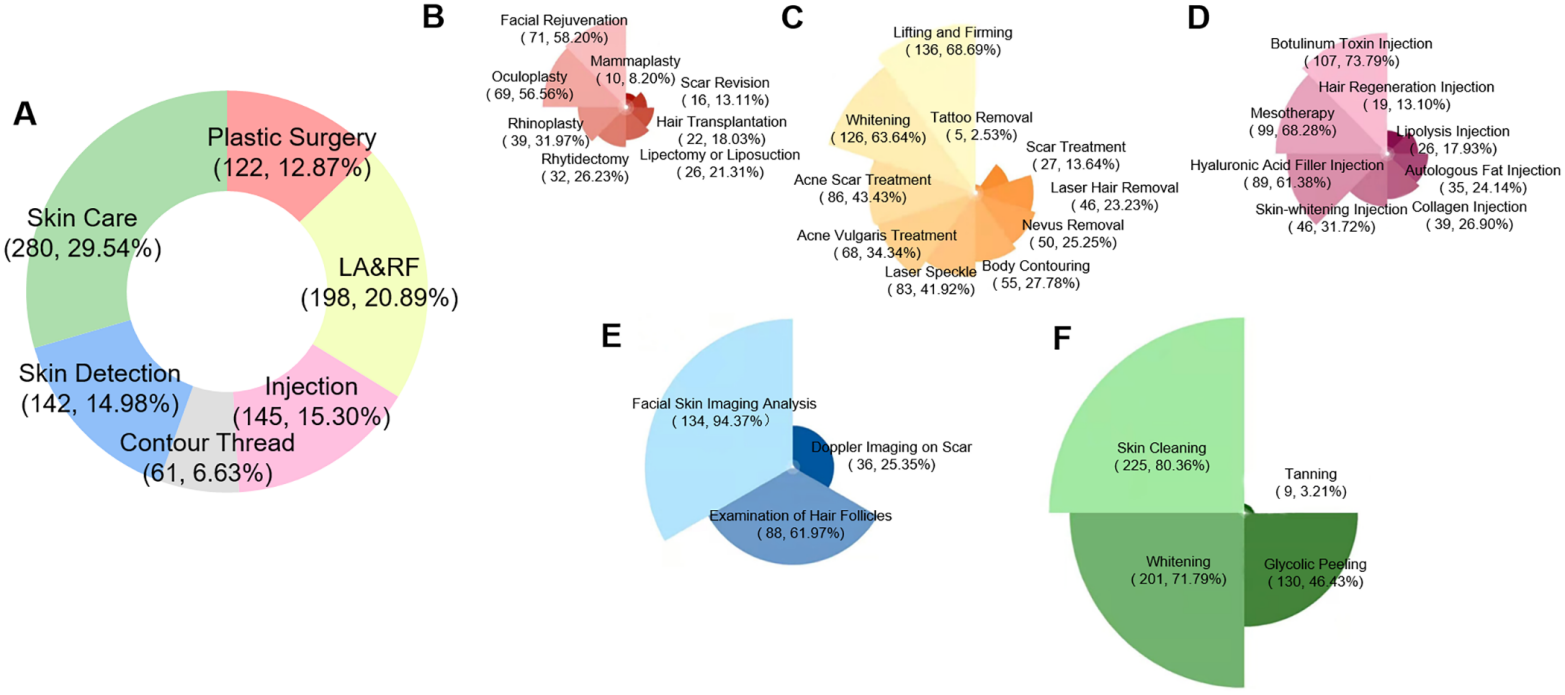
Participants' preferred types of cosmetic treatments since the implementation of PHSMs. The size of each fan blade represents the numbers in each group of the Nightingale rose diagram. The red, yellow, pink, blue, and green fan blades represent the subtypes of plastic surgery, laser and radiofrequency, injection, skin detection, and skin care, respectively. (A) The preferred subtypes of treatments for all participants with the intention of undergoing plastic surgery. (B) The preferred subtypes of treatments for all participants with the intention of undergoing laser and radiofrequency treatment. (C) The preferred subtypes of treatments for all participants with the intention of undergoing injections. (D) The preferred subtypes of medical practices for all participants with the intention of undergoing skin detection. (E) The preferred subtypes of medical practices for all participants with the intention of undergoing skin care. LA&RF, laser and radiofrequency.

To explore the preferred types of cosmetic treatments among participants, we classified 35 items into six major types: plastic surgery (12.87%), laser and radiofrequency (20.87%), injections (15.30%), contour threading (6.43%), skin detection for skin status (14.98%), and skin care (29.54%) (Figure 3A). Over 50% of participants intending to undergo plastic surgery planned to undergo oculoplasty and facial rejuvenation (Figure 3B), and over 60% of participants were interested in skin whitening as well as lifting and firming in the “laser and radiofrequency” category (Figure 3C). Hyaluronic acid filler, botulinum toxin, and mesotherapy were the most commonly selected injection treatments, with proportions of 61.38%, 73.79%, and 68.28%, respectively (Figure 3D). Furthermore, facial skin imaging analysis, hair follicle examination, skin cleaning, and whitening were the main demands in terms of skin detection and skin care, with proportions of over 60% (Figure 3E,F). The preferred options and factors in the acceptance of cosmetic treatments and the preferred types of treatments during and after PHSMs are demonstrated in Tables S5–S7. Except for the higher proportion preferring skin detection after PHSMs (*p* = 0.027), no significant differences were detected in the proportions of the other items during and after PHSMs.

## 4 Discussion

### 4.1 Main Findings and Interpretation

Since the COVID‐19 pandemic, some other epidemic diseases, such as influenza A and dengue fever, which are characterized by regional aggregation and high transmissibility/pathogenicity, have prevailed in numerous countries worldwide [3, 16]. Evidence‐based public health practices identified that PHSMs could control the prevalence of various viral infections by reducing contact frequency with the carriers of the virus [17]. However, PHSMs delayed and decreased various types of elective surgery as well as non‐surgical treatments to varying degrees, including cosmetic treatments, while the planned medical visits of patients were also affected by PHSMs [18, 19]. For the first time, our work systematically evaluated the impact of PHSMs on the medical practices of and public preferences for cosmetic treatments in the context of the COVID‐19 pandemic to provide a practical basis for the development of cosmetic treatments during and after future PHSMs.

By analyzing the clinical data on cosmetic treatments from three public and three private hospitals from October 1, 2021, to May 30, 2022, and calculating the composition of surgical and non‐surgical treatments in public and private hospitals, the aftereffects of PHSMs on treatment volumes and proportions in the two types of hospitals demonstrated different time–series characteristics (Figure 1A–C). When faced with PHSMs, increases in the volume of surgical treatments after the measures occurred sooner than with non‐surgical procedures, especially in public hospitals, which were dominated by surgical treatments. Therefore, the impact of the duration of PHSMs on treatment volumes in public hospitals was clearly shorter than that in private hospitals. For the average weekly volumes in different periods, in public hospitals after PHSMs, there was always a sharp increase related to highly impulsive patient consumption, particularly in terms of plastic surgery, and surgical volumes after PHSMs were always higher than those before PHSMs. However, non‐surgical treatments were mainly implemented in private hospitals, and the magnitude of the increase in the volume of non‐surgical treatments after PHSMs was lower than with plastic surgery, which equaled the pre‐PHSM volume. Therefore, the negative impact of PHSM implementation on public hospitals during the COVID‐19 pandemic was much lower than the impact on private hospitals; PHSMs could even be a new growth point for surgical treatments in public hospitals.

Public health and social measures entail restrictions on travel and movement and on social and physical distancing. They are significantly associated with anxiety, perceived stress, and cognitive insights in the general population [17, 20, 21], with such psychological states being proven to influence both patients' plans and the postoperative outcomes of cosmetic treatments [22, 23]. Combining the investigation of the relationships among participants' anxiety states, self‐perceived appearance, and intention to undergo cosmetic treatment, we inferred that PHSMs were likely to lead to states of anxiety in the population. The negative self‐perception of appearance and the increased intention to undergo cosmetic treatments might be caused by increased states of anxiety during PHSMs, which could explain the growing proportion with a high intention of undergoing cosmetic treatments during PHSMs and the impulsive consumption of cosmetic treatments after PHSMs. When meeting patients in clinics after PHSMs, doctors are responsible for reducing patients' anxiety to avoid unreasonable, excessive cosmetic treatments.

Besides psychological factors, some external causes, such as social factors, also influence people's decisions to undergo cosmetic treatments [24, 25]. Through our preliminary survey, we selected the influential factors for further investigation in terms of choosing medical institutions, selecting doctors, expected costs, and anticipated recovery time, all of which were vital factors in the treatment choices of patients. In the post‐COVID‐19 era, medical institutions should try their best to attract and retain talented doctors with advanced professional titles so that their increased competitiveness is rewarded by attracting more patients for both doctors and highly professional doctor‐owned institutions. Moreover, the cost of procedures should be revised, as costs of 1000–10 000 CNY were accepted by 71.72% of participants during and after PHSMs, and a series of medical treatments should be completed within 3–14 days to satisfy most patients' desire to return to normal life.

The final section of the questionnaire aimed to explore public demands during and after PHSMs to guide the allocation of healthcare resources in the post‐COVID‐19 era. Interestingly, despite the differences in various types of treatments in different periods, no significant differences were detected in the proportion of treatment types, but a higher proportion was noted in terms of skin detection after PHSMs (Tables S6 and S7). Therefore, after the implementation of PHSMs in COVID‐19, public demand for the different types of cosmetic treatments has been stable. Furthermore, we found that participants' high‐ranked preferences in terms of the classified items were partly different from the variation trends of real medical practices during and after PHSMs. As most elective treatments, especially for plastic surgery, were postponed during the COVID‐19 pandemic [26], treatments should be implemented with hysteresis in response to the demands of the population. The results of our questionnaire study could compensate for this delay by reflecting the present demand for cosmetic treatments.

Considering the high proportion of various demands, medical institutions should take measures to improve the capability of medical services in skin care and skin detection, particularly in private hospitals dominated by non‐surgical treatments. Public hospitals should continue to develop their advantages in plastic surgery, while the increased demand for non‐surgical treatments after PHSMs means that the allocation of medical resources and doctors should be adjusted according to public demands.

### 4.2 Limitations and Conclusion

The medical records used in our retrospective study were from six hospitals in Xi'an, so further investigation is needed to explore the actual medical practices in hospitals across the world during COVID‐19. Furthermore, due to the capacity limitations of medical treatment, none of the departments of plastic surgery and cosmetic dermatology in the six hospitals could conduct all the items listed in our research. On the other hand, considering the practical feasibility of the project, our questionnaire‐based study selected only two time points for follow‐up analysis, and the validated questionnaires we collected were the maximum sample size that we could obtain. Despite the actual number of validated questionnaires being over the expected minimum participant sample size, including more samples in more time points, especially before the implementation of PHSMs, will be necessary for future research.

In conclusion, this work has both present and long‐term guiding significance in different dimensions. In the context of the COVID‐19 pandemic, our multi‐center retrospective study revealed the time–series characteristics before, during, and after PHSMs and demonstrated the different aftereffects of PHSMs on surgical and non‐surgical treatments in public and private hospitals. Therefore, in terms of long‐term guiding significance, our work could provide a reference for the development of plastic surgery and cosmetic dermatology when confronted by PHSMs caused by other situations in the future. The subsequent questionnaire‐based study elucidated the impact of psychological states during PHSMs on the public's intention to undergo cosmetic treatment. More importantly, we systematically analyzed the public's preference for cosmetic treatment since the implementation of PHSM. Therefore, as the present study offers guiding significance in the post‐COVID‐19 era, demand and supply‐based operating modes could be adjusted to attract more patients to both hospitals and doctors and optimize the resources allocated to cosmetic treatments.

## Author Contributions

B.S., Z.Y., and T.L. contributed to the study and the design of the questionnaire. Z.L. and L.Z. assisted in collecting the medical records from multiple medical institutions. Z.L. and Y.Z. contributed to the questionnaire's delivery and collection. Z.L. and T.L. assisted in questionnaire pre‐testing and quality control of the valid questionnaires. Z.L. and T.L. contributed to the data analysis. Z.L. conducted data visualization. Z.L. Y.Z. and B.S. wrote and revised the manuscript. All authors read and approved the final manuscript.

## Ethics Statement

This study is in accordance with the Declaration of Helsinki. All participants signed inform consent.

Human Ethics: This study was approved by the ethics committee of Xijing Hospital of the Fourth Military Medical University (KY2022006‐D‐2).

## Consent

This provision is not applicable for this study.

## Conflicts of Interest

The authors declare no conflicts of interest.

## Supporting information


Table S1–S8.

## Data Availability

Data are available on https://www.jianguoyun.com/p/DUEX1WsQuaiFChjs57UFIAA.
