# Supplementary material for: Impact of Public Health and Social Measures on Cosmetic Treatments in the COVID‐19 Pandemic: A Retrospective Multi‐Center Study Combined With a Questionnaire‐Based Cross‐Sectional Study
Source: J Cosmet Dermatol. 2024 Sep 24;23(12):3800–8. doi: 10.1111/jocd.16563 (PMC11626295; doi:10.1111/jocd.16563)
Supplement: Supplementary file 1 — Table S1–S8. [file JOCD-23--s001.docx]

| Supplementary Table 1. Main content of the questionnaire |
| --- |
|  |

**Part 1. Basic information**

(1) Your gender is __________.

A. Female; B. Male

(2) Your age is __________.

A. 18–30 years; B. 30–50 years; C. ≥50 years

(3) Your marital status is __________.

A. Married; B. Unmarried

(4) Your salary is about __________ a month.

A. <5k CNY; B. 5–10k CNY; C. ≥10k CNY

(5) Do the present public health and social measures (PHSMs) restrict your daily travel [habits] and life?

A. Yes; B. No

**Part 2. Anxiety states and self-perception of appearance**

(6) Do you feel nervous or anxious?

A. A little of the time; B. Some of the time; C. A good part of the time; D. Most of the time

(7) Do you feel panicky or restless?

A. A little of the time; B. Some of the time; C. A good part of the time; D. Most of the time

(8) Do you have difficulty in falling asleep or getting a good night’s rest?

A. A little of the time; B. Some of the time; C. A good part of the time; D. Most of the time

(9) Do you feel anxious because of your appearance?

A. A little of the time; B. Some of the time; C. A good part of the time; D. Most of the time

(10) How do you think your appearance has changed recently?

A. Better; B. Worse; C. No obvious change

(11) If your answer to item 10 was “better” or “worse,” please answer this question: In your opinion, which aspects of your appearance have changed recently?

A. Skin texture; B. Hair density; C. Stature; D. Cutis laxa

(12) Do you have any plans to undergo cosmetic treatments?

A. Yes; B. No

(13) How do PHSMs affect your intention to undergo cosmetic treatment?

A. Increase the intention; B. Decrease the intention; C. No influence

**Part 4. Factors influencing the choice of medical institutions and doctors**

(14) Which of the following medical institutions will you choose for cosmetic treatment?

A. Public hospitals; B. Private hospitals; C. Skin management centers

(15) Which of the following factors do you consider when you choose medical institutions? (multiple selection)

A. Comprehensive strength in a specialized field; B. Media publicity; C. Priority is given to a preferred doctor; D. Recommendation from acquaintance; E. Low cost; F. Convenient location

(16) Which of the following factors do you consider when choosing your attending doctor? (multiple selection)

A. Comprehensive strength of the institution in which the doctor works; B. Professional title; C. Media publicity; D. Recommendation from acquaintance; E. Low cost

(17) How long is your expected recovery time after medical cosmetology?

A. <3 days; B. 3–5 days; C. 7–14 days; D. ≥14 days

(18) How much is your expected cost of medical cosmetology?

A. <1k CNY; B. 1–3k CNY; C. 3–5k CNY; D. 5–10k CNY; E. ≥10k CNY

**Part 5. Public preference for types of cosmetic treatments**

(19) Which of the following types of cosmetic treatments are you interested in? (multiple selection)

A. Plastic surgery; B. Laser and radiofrequency; C. Injections; D. Contour threading; E. Skin detection; F. Skin care

(20) If your answer to item 19 was “plastic surgery,” please answer this question: Which of the following surgeries are you interested in? (multiple selection)

A. Oculoplasty; B. Rhinoplasty; C. Facial rejuvenation; D. Hair transplantation; E. Mammaplasty; F. Lipectomy or liposuction; G. Rhytidectomy; H. Scar revision

(21) If your answer to item 19 was “laser and radiofrequency,” please answer this question: Which of the following aspects are you interested in in terms of laser and radiofrequency? (multiple selection)

A. Acne vulgaris treatment; B. Acne scar treatment; C. Laser speckle; D. Laser hair removal; E. Whitening; F. Lifting and firming; G. Skin anti-aging; H. Nevus removal; I. Scar treatment; J. Body contouring

(22) If your answer to item 19 was “injections,” please answer this question: Which of the following injection strategies are you interested in? (multiple selection)

A. Hyaluronic acid filler injection; B. Botulinum toxin injection; C. Mesotherapy; D. Autologous fat injection; E. Collagen injection; F. Lipolysis injection; G. Hair regeneration injection; H. Skin-whitening injection

(23) If your answer to item 19 was “skin detection,” please answer this question: Which of the following items of skin detection are you interested in? (multiple selection)

A. Facial skin imaging analysis; B. Examination of hair follicles; C. Doppler imaging of scar

(24) If your answer to item 19 was “skin care,” please answer this question: Which of the following items of skin care are you interested in? (multiple selection)

A. Whitening; B. Tanning; C. Glycolic peeling; D. Skin cleaning; E. Micro bubble; F. SPA

(25) Do you completely understand the above questions?

A. Yes; B. No

|  |  |  |  |  |  |  |  |
| --- | --- | --- | --- | --- | --- | --- | --- |

**Supplementary Table 2. Time-dependent variations in average weekly volumes and proportion of treatments in three public hospitals**

|  | **Before PHSMs *n* (%)** |  | **During PHSMs *n* (%)** |  | **1 Month After PHSMs *n* (%)** |  | **2 Months After PHSMs *n* (%)** |  | **Normal Stage *n* (%)** |
| --- | --- | --- | --- | --- | --- | --- | --- | --- | --- |
| **Surgical Treatments** |  |  |  |  |  |  |  |  |  |
| Oculoplasty | 1,446 (58.12) |  | 304 (71.03) |  | 685 (49.85) |  | 431 (44.11) |  | 1,413 (51.46) |
| Otoplasty | 82 (3.30) |  | 11 (2.57) |  | 41 (2.98) |  | 26 (2.66) |  | 60 (2.18) |
| Rhinoplasty | 96 (3.86) |  | 12 (2.80) |  | 47 (3.42) |  | 29 (2.97) |  | 111 (4.04) |
| Mammaplasty | 42 (1.69) |  | 4 (0.93) |  | 18 (1.31) |  | 34 (3.48) |  | 65 (2.37) |
| Labiaplasty | 30 (1.21) |  | 5 (1.17) |  | 17 (1.24) |  | 12 (1.23) |  | 30 (1.09) |
| Maxillofacial Surgery/Facial Osteoplasty | 25 (1.00) |  | 3 (0.70) |  | 8 (0.58) |  | 23 (2.35) |  | 64 (2.33) |
| Rhytidectomy | 4 (0.16) |  | 1 (0.23) |  | 2 (0.15) |  | 4 (0.41) |  | 6 (0.22) |
| Liposuction | 8 (0.32) |  | 0 (0.00) |  | 7 (0.51) |  | 14 (1.43) |  | 27 (0.98) |
| Facial Lipofilling | 35 (1.41) |  | 4 (0.93) |  | 12 (0.87) |  | 14 (1.43) |  | 70 (2.55) |
| Abdominal Wall Reconstruction | 2 (0.08) |  | 0 (0.00) |  | 2 (0.15) |  | 1 (0.10) |  | 10 (0.36) |
| Hair Transplantation | 19 (0.76) |  | 1 (0.23) |  | 6 (0.44) |  | 11 (1.13) |  | 20 (0.73) |
| Genital Cosmetic Surgery | 52 (2.09) |  | 7 (1.64) |  | 22 (1.60) |  | 22 (2.25) |  | 45 (1.64) |
| Scar Revision | 74 (2.97) |  | 3 (0.70) |  | 97 (7.06) |  | 47 (4.81) |  | 82 (2.99) |
| ^1^Other | 573 (23.03) |  | 73 (17.06) |  | 410 (29.84) |  | 309 (31.63) |  | 743 (27.06) |
| Total | 2,488 (100.00) |  | 428 (100.00) |  | 1,374 (100.00) |  | 977 (100.00) |  | 2,746 (100.00) |
| **Non-surgical Treatments** |  |  |  |  |  |  |  |  |  |
| Laser and Radiofrequency | 16 (1.22) |  | 4 (2.08) |  | 53 (7.68) |  | 35 (5.20) |  | 50 (3.98) |
| Mesotherapy | 806 (61.39) |  | 147 (76.56) |  | 359 (52.03) |  | 396 (58.84) |  | 672 (53.50) |
| Botulinum Toxin Injection | 255 (19.42) |  | 4 (2.08) |  | 201 (29.13) |  | 122 (18.13) |  | 358 (28.50) |
| Hyaluronic Acid Filler Injection | 236 (17.97) |  | 37 (19.27) |  | 77 (11.16) |  | 120 (17.83) |  | 176 (14.01) |
| Collagen Injection | 0 (0.00) |  | 0 (0.00) |  | 0 (0.00) |  | 0 (0.00) |  | 0 (0.00) |
| Contour Threading | 0 (0.00) |  | 0 (0.00) |  | 0 (0.00) |  | 0 (0.00) |  | 0 (0.00) |
| Total | 1,313 (100.00) |  | 192 (100.00) |  | 690 (100.00) |  | 673 (100.00) |  | 1,256 (100.00) |

*^1^Other: Other types of surgical treatments in public hospitals*

*Abbreviation: PHSMs: Public health and social measures*

**Supplementary Table 3. Time-dependent variations in average weekly volumes and proportion of treatments in private hospitals**

|  | **Before PHSMs *n* (%)** |  | **During PHSMs *n* (%)** |  | **1 Month After PHSMs *n* (%)** |  | **2 Months After PHSMs *n* (%)** |  | **Normal Stage *n* (%)** |
| --- | --- | --- | --- | --- | --- | --- | --- | --- | --- |
| **Surgical Treatments** |  |  |  |  |  |  |  |  |  |
| Oculoplasty | 1,281 (36.25) |  | 58 (45.31) |  | 424 (31.62) |  | 505 (35.44) |  | 972 (35.19) |
| Otoplasty | 0 (0.00) |  | 0 (0.00) |  | 0 (0.00) |  | 0 (0.00) |  | 0 (0.00) |
| Rhinoplasty | 371 (10.50) |  | 19 (14.81) |  | 149 (11.11) |  | 148 (10.39) |  | 229 (8.29) |
| Mammaplasty | 108 (3.06) |  | 8 (6.25) |  | 40 (2.98) |  | 61 (4.28) |  | 122 (4.42) |
| Labiaplasty | 19 (0.54) |  | 1 (0.78) |  | 4 (0.30) |  | 4 (0.28) |  | 4 (0.14) |
| Maxillofacial Surgery/Facial Osteoplasty | 49 (1.39) |  | 1 (0.78) |  | 14 (1.04) |  | 14 (0.98) |  | 33 (1.19) |
| Rhytidectomy | 1 (0.03) |  | 0 (0.00) |  | 1 (0.07) |  | 0 (0.00) |  | 2 (0.07) |
| Liposuction | 518 (14.66) |  | 29 (22.66) |  | 193 (14.39) |  | 293 (20.56) |  | 528 (19.12) |
| Facial Lipofilling | 383 (10.84) |  | 9 (7.03) |  | 157 (11.71) |  | 146 (10.25) |  | 219 (7.93) |
| Abdominal Wall Reconstruction | 0 (0.00) |  | 0 (0.00) |  | 0 (0.00) |  | 0 (0.00) |  | 0 (0.00) |
| Hair Transplantation | 56 (1.58) |  | 2 (1.56) |  | 13 (0.97) |  | 21 (1.47) |  | 40 (1.45) |
| Genital Cosmetic Surgery | 18 (0.51) |  | 0 (0.00) |  | 6 (0.45) |  | 3 (0.21) |  | 30 (1.09) |
| Scar Revision | 274 (7.75) |  | 0 (0.00) |  | 154 (11.48) |  | 58 (4.07) |  | 257 (9.30) |
| ^1^Other | 456 (12.90) |  | 1 (0.78) |  | 186 (13.87) |  | 172 (12.07) |  | 326 (11.80) |
| Total | 3,534 (100.00) |  | 128 (100.00) |  | 1,341 (100.00) |  | 1,425 (100.00) |  | 2,762 (100.00) |
| **Non-surgical Treatments** |  |  |  |  |  |  |  |  |  |
| Laser and Radiofrequency | 17,133 (35.35) |  | 340 (16.35) |  | 4,138 (28.08) |  | 4,839 (29.69) |  | 11,263 (29.54) |
| Mesotherapy | 11,966 (24.69) |  | 597 (28.72) |  | 3,595 (24.39) |  | 4,088 (25.08) |  | 8,850 (23.21) |
| Botulinum Toxin Injection | 13,409 (27.66) |  | 897 (43.15) |  | 4,818 (32.69) |  | 5,192 (31.86) |  | 13,253 (34.76) |
| Hyaluronic Acid Filler Injection | 5,537 (11.42) |  | 214 (10.29) |  | 2,064 (14.01) |  | 2,071 (12.71) |  | 4,511 (11.83) |
| Collagen Injection | 224 (0.46) |  | 25 (1.20) |  | 83 (0.56) |  | 77 (0.47) |  | 200 (0.52) |
| Contour Threading | 202 (0.42) |  | 6 (0.29) |  | 39 (0.26) |  | 30 (0.18) |  | 45 (0.12) |
| Total | 48,471(100.00) |  | 2,079 (100.00) |  | 14,737 (100.00) |  | 16,297 (100.00) |  | 38,122(100.0) |

*^1^Other: Other types of surgical treatments in private hospitals*

**Supplementary Table 4. The self-perceived appearance of participants during and after PHSMs**

|  | **Better Appearance** | |  | **Worse Appearance** | |  |
| --- | --- | --- | --- | --- | --- | --- |
|  | **PHSMs *n* (%)** | **After PHSMs *n* (%)** | ***P* Value** | **PHSMs *n* (%)** | **After PHSMs *n* (%)** | ***P* Value** |
| **Skin Texture** | 27 (5.33) | 20 (3.91) | 0.283 | 129 (25.44) | 100 (19.57) | 0.025 |
| **Hair Density** | 10 (1.97) | 9 (1.76) | 0.496 | 53 (10.45) | 65 (12.72) | 0.259 |
| **Stature** | 21 (4.14) | 19 (3.72) | 0.728 | 93 (18.34) | 89 (17.42) | 0.700 |
| **Cutis Laxa** | 9 (1.78) | 8 (1.57) | 0.794 | 59 (11.64) | 56 (10.96) | 0.733 |

*Abbreviation: PHSMs: Public health and social measures*

**Supplementary Table 5. Public preference in undergoing cosmetic treatments during and after PHSMs**

|  | **PHSMs *n* (%)** | **After PHSMs *n* (%)** | ***P* Value** |
| --- | --- | --- | --- |
|  |  |  |  |
| **Preference for Medical Institutions** |  |  | 0.331 |
| Public Hospital | 173 (79.72) | 135 (75.42) |  |
| Private Hospital | 17 (7.83) | 22 (12.29) |  |
| Skin Management Center | 27 (12.44) | 22 (12.29) |  |
| **Factors Influencing Choice of Medical Institution** |  |  |  |
| Comprehensive Strength in Specialized Field | 151 (69.59) | 134 (74.86) | 0.262 |
| Media Publicity | 9 (4.15) | 12 (6.70) | 0.270 |
| Priority Given to the Preferred Doctor | 140 (64.52) | 112 (62.57) | 0.753 |
| Recommendation from Acquaintance | 78 (35.94) | 80 (44.69) | 0.081 |
| Low Cost | 65 (29.95) | 67 (37.43) | 0.134 |
| Convenient Location | 43 (19.82) | 39 (21.79) | 0.709 |
| **Factors Influencing Choice of Doctor** |  |  |  |
| Comprehensive Strength of the Institution in Which the Doctor Works | 159 (73.27) | 138 (77.09) | 0.415 |
| Professional Title | 164 (75.58) | 127 (70.95) | 0.306 |
| Media Publicity | 96 (44.24) | 89 (49.72) | 0.312 |
| Recommendation from Acquaintance | 89 (41.01) | 83 (46.37) | 0.309 |
| Low Cost | 43 (19.82) | 32 (17.88) | 0.699 |
| **Expected Recovery Time** |  |  | 0.216 |
| <3 days | 45 (20.74) | 27 (15.08) |  |
| 3–7 days | 88 (40.55) | 82 (45.81) |  |
| 7–14 days | 60 (27.65) | 57 (31.84) |  |
| ≥14 days | 24 (11.06) | 13 (7.26) |  |
| **Expected Expenses (CNY)** |  |  | 0.161 |
| <1k | 28 (12.90) | 23 (12.85) |  |
| 1–3k | 57 (26.27) | 62 (34.64) |  |
| 3–5k | 53 (24.42) | 28 (15.64) |  |
| 5–10k | 48 (22.12) | 36 (20.11) |  |
| ≥10k | 31 (14.29) | 30 (16.76) |  |

*Abbreviations: PHSMs: Public health and social measures; CNY: Chinese yuan*

**Supplementary Table 6. Preferred treatment types of all participants with the intention of undergoing cosmetic treatments**

|  | **PHSMs**  ***n* (%)** | **After PHSMs *n* (%)** | ***P* Value***** |
| --- | --- | --- | --- |
|  |  |  |  |
| Plastic Surgery | 62 (28.57) | 60 (33.52) | 0.325 |
| Laser and Radiofrequency | 108 (49.77) | 90 (50.28) | 1.000 |
| Injection | 85 (39.17) | 60 (33.52) | 0.251 |
| Contour Threading | 32 (14.75) | 29 (16.20) | 0.780 |
| Skin Detection | 67 (30.88) | 75 (41.90) | 0.027 |
| Skin care | 147 (67.74) | 133 (74.30) | 0.183 |

*Abbreviation: PHSMs: Public health and social measures*

|  | **PHSMs  *n* (%)** | **After PHSMs  *n* (%)** | ***P* Value** |
| --- | --- | --- | --- |
| **Plastic Surgery** |  |  |  |
| Oculoplasty | 35 (56.45) | 34 (56.67) | 1.000 |
| Rhinoplasty | 16 (25.81) | 23 (38.33) | 0.175 |
| Facial Rejuvenation | 34 (54.84) | 37 (61.67) | 0.468 |
| Hair Transplantation | 11 (17.74) | 11 (18.33) | 1.000 |
| Mammaplasty | 6 (9.68) | 4 (6.67) | 0.744 |
| Lipectomy or Liposuction | 13 (20.97) | 13 (21.67) | 1.000 |
| Rhytidectomy | 13 (20.97) | 19 (31.67) | 0.219 |
| Scar Revision | 7 (11.29) | 9 (15.00) | 0.600 |
| **Laser and Radiofrequency** |  |  |  |
| Acne Vulgaris Treatment | 35 (32.41) | 33 (36.67) | 0.551 |
| Acne Scar Treatment | 50 (46.30) | 36 (40.00) | 0.391 |
| Laser Speckle | 45 (41.67) | 38 (42.22) | 1.000 |
| Laser Hair Removal | 22 (20.37) | 24 (26.67) | 0.315 |
| Whitening | 70 (64.81) | 56 (62.22) | 0.767 |
| Lifting and Firming | 69 (63.89) | 67 (74.44) | 0.125 |
| Tattoo Removal | 3 (2.78) | 2 (2.22) | 1.000 |
| Nevus Removal | 30 (27.78) | 20 (22.22) | 0.414 |
| Scar Treatment | 18 (16.67) | 9 (10.00) | 0.214 |
| Body Contouring | 32 (29.63) | 23 (25.56) | 0.633 |
| **Injection** |  |  |  |
| Hyaluronic Acid Filler Injection | 54 (63.53) | 35 (58.33) | 0.604 |
| Botulinum Toxin Injection | 62 (72.94) | 45 (75.00) | 0.849 |
| Mesotherapy | 55 (64.71) | 44 (73.33) | 0.284 |
| Autologous Fat Injection | 24 (28.24) | 11 (18.33) | 0.237 |
| Collagen Injection | 26 (30.59) | 13 (21.67) | 0.259 |
| Lipolysis Injection | 15 (17.65) | 11 (18.33) | 1.000 |
| Hair Regeneration Injection | 13 (15.29) | 6 (10.00) | 0.456 |
| Skin-whitening Injection | 28 (32.94) | 18 (30.00) | 0.722 |
| **Skin Detection** |  |  |  |
| Facial Skin Imaging Analysis | 62 (92.54) | 72 (96.00) | 0.476 |
| Examination of Hair Follicles | 43 (64.18) | 45 (60.00) | 0.729 |
| Doppler Imaging of Scar | 18 (26.87) | 18 (24.00) | 0.704 |
| **Skin care** |  |  |  |
| Whitening | 103 (70.07) | 98 (73.68) | 0.510 |
| Tanning | 4 (2.72) | 5 (3.76) | 0.740 |
| Glycolic Peeling | 71 (48.30) | 59 (44.36) | 0.549 |
| Skin Cleaning | 115 (78.23) | 107 (80.45) | 0.661 |

**Supplementary Table 7. Preferred subtypes of treatments for participants with the intention of undergoing cosmetic treatment**

*Abbreviation: PHSMs: Public health and social measures*

**Supplementary Table 8. Relationships in the cross-sectional study between anxiety state and self-perception of appearance and intention to undergo cosmetic treatment**

|  | **Better in Appearance *n* (%)** | **Worse in Appearance *n* (%)** | **No Change *n* (%)** | ***P* Value** | **CT Intention *n* (%)** | **No CT intention *n* (%)** | ***P* Value** | **Increased**  **CT Intention *n* (%)** | **Decreased**  **CT Intention *n* (%)** | **No Influence on CT Intention *n* (%)** | ***P* Value** |
| --- | --- | --- | --- | --- | --- | --- | --- | --- | --- | --- | --- |
| **^1^Question 6** |  |  |  |  |  |  |  |  |  |  |  |
| No Anxiety | 47 (9.00) | 70 (13.40) | 404 (77.50) |  | 176 (44.44) | 345 (55.47) |  | 39 (31.45) | 97 (51.05) | 385 (54.69) |  |
| Mild Anxiety | 25 (6.70) | 145 (38.80) | 204 (54.50) |  | 168 (42.42) | 206 (33.12) |  | 46 (37.10) | 72 (37.89) | 256 (36.36) |  |
| Moderate Anxiety | 0 (0.00) | 59 (62.80) | 35 (37.20) |  | 41 (10.35) | 53 (8.52) |  | 30 (24.19) | 15 (7.89) | 49 (6.96) |  |
| Severe Anxiety | 0 (0.00) | 22 (75.90) | 7 (24.10) |  | 11 (2.78) | 18 (2.89) |  | 9 (7.26) | 6 (3.16) | 49 (1.99) |  |
| Average Rank | 415.53 | 671.14 | 445.23 | <0.001 | 542.05 | 488.78 | 0.002 | 645.81 | 507.84 | 485.94 | <0.001 |
| **^2^Question 7** |  |  |  |  |  |  |  |  |  |  |  |
| No Anxiety | 55 (10.50) | 63 (12.10) | 404 (77.40) |  | 172 (43.43) | 350 (56.27) |  | 34 (27.42) | 99 (52.11) | 389 (55.26) |  |
| Mild Anxiety | 17 (4.40) | 131 (34.10) | 236 (61.50) |  | 172 (43.43) | 212 (34.08) |  | 50 (40.32) | 78 (41.05) | 256 (36.36) |  |
| Moderate Anxiety | 0 (0.00) | 76 (88.40) | 10 (11.60) |  | 42 (10.61) | 44 (7.07) |  | 30 (24.19) | 7 (3.68) | 49 (6.96) |  |
| Severe Anxiety | 0 (0.00) | 26 (100.00) | 0 (0.00) |  | 10 (2.53) | 16 (2.57) |  | 10 (8.06) | 6 (3.16) | 10 (1.42) |  |
| Average Rank | 367.72 | 703.99 | 435.67 | <0.001 | 550.02 | 483.71 | <0.001 | 670.61 | 496.31 | 484.68 | <0.001 |
| **^3^Question 8** |  |  |  |  |  |  |  |  |  |  |  |
| No Anxiety | 58 (11.20) | 69 (13.30) | 391 (75.50) |  | 182 (45.96) | 336 (54.02) |  | 37 (29.84) | 98 (51.58) | 383 (54.40) |  |
| Mild Anxiety | 14 (3.80) | 109 (29.30) | 249 (66.90) |  | 154 (38.89) | 218 (35.05) |  | 41 (33.06) | 75 (39.47) | 256 (36.36) |  |
| Moderate Anxiety | 0 (0.00) | 92 (94.80) | 5 (5.20) |  | 47 (11.87) | 50 (8.04) |  | 36 (29.03) | 11 (5.79) | 50 (7.10) |  |
| Severe Anxiety | 0 (0.00) | 26 (83.90) | 5 (16.10) |  | 13 (3.28) | 18 (2.89) |  | 10 (8.06) | 6 (3.16) | 15 (2.13) |  |
| Average Rank | 345.33 | 699.88 | 440.01 | <0.001 | 537.61 | 491.6 | 0.007 | 663.87 | 497.98 | 485.42 | <0.001 |
| **^4^Question 9** |  |  |  |  |  |  |  |  |  |  |  |
| No Anxiety | 60 (12.30) | 48 (9.80) | 381 (77.90) |  | 148 (37.37) | 341 (54.82) |  | 28 (22.58) | 89 (46.84) | 372 (52.84) |  |
| Mild Anxiety | 12 (3.20) | 104 (27.70) | 260 (69.10) |  | 170 (42.93) | 206 (33.12) |  | 39 (31.45) | 84 (44.21) | 253 (35.94) |  |
| Moderate Anxiety | 0 (0.00) | 105 (94.60) | 6 (5.40) |  | 61 (15.40) | 50 (8.04) |  | 42 (33.87) | 10 (5.26) | 59 (8.38) |  |
| Severe Anxiety | 0 (0.00) | 39 (92.90) | 3 (7.10) |  | 17 (4.29) | 25 (4.02) |  | 15 (12.10) | 7 (3.68) | 20 (2.84) |  |
| Average Rank | 316.92 | 734.97 | 427.29 | <0.001 | 567.10 | 472.83 | <0.001 | 701.02 | 499.51 | 478.46 | <0.001 |

*^1^Question 6: Do you feel nervous and anxious?*

*^2^Question 7: Do you feel panicky or restless?*

*^3^Question 8: Do you have difficulty in falling asleep or getting a good night's rest?*

*^4^Question 9:* *Do you feel anxious because of your appearance?*

*Abbreviation: CT Intention: Intention to undergo cosmetic treatment.*

*Scores 1–4 were assignments of “No Anxiety,” “Mild Anxiety,” “Moderate Anxiety,” and “Severe Anxiety,” respectively.*

*Average ranks were calculated using the Wilcoxon–Mann–Whitney test and the Kruskal–Wallis test.*
